# Airway Management Challenges in Three Cases of Vallecular Cysts

**DOI:** 10.7759/cureus.90213

**Published:** 2025-08-16

**Authors:** Dharani Manthirasalam, Gayathri Ramesh

**Affiliations:** 1 Anaesthesiology, Sree Balaji Medical College and Hospital, Chennai, IND

**Keywords:** awake fibreoptic intubation (afoi), cyst rupture, mucous retention cyst, nasal packing, vallecular cyst

## Abstract

Vallecular cysts, though rare in adults, can pose significant challenges to anesthetic management due to their location near the airway and potential for life-threatening complications such as airway obstruction, aspiration, or cyst rupture during intubation. This case series presents three adult male patients who reported with symptoms ranging from dysphagia and voice changes to globus sensation, each diagnosed with vallecular cysts via imaging and endoscopic evaluation. All patients underwent surgical excision under general anesthesia following awake fiberoptic intubation (AFOI) to avoid airway compromise. Preoperative preparation involved comprehensive airway assessment, regional airway blocks, topical anesthesia, and conscious sedation. Anesthesia was successfully maintained with balanced agents, and each surgery concluded without intraoperative or postoperative complications. These cases underscore the importance of meticulous preoperative planning, individualized airway strategies, and use of AFOI in patients with potentially obstructive vallecular cysts. Such tailored approaches are critical for safe and effective anesthetic management in these high-risk scenarios.

## Introduction

Vallecular cysts are rare, mucus-retention lesions arising from the base of the tongue or epiglottis, often causing airway obstruction, dysphagia, or stridor [1]. While more common in infants, they can present in adults with significant anesthetic challenges due to the risk of cyst rupture, aspiration, and difficult airway management [2]. The primary concern during surgical intervention is securing the airway without compromising cyst integrity, as rupture can lead to life-threatening complications such as laryngospasm, anaphylaxis, or pneumonia [3].

Awake fiberoptic intubation (AFOI) is the gold standard for managing such cases, as it minimizes trauma while maintaining spontaneous ventilation [4]. However, alternative techniques, including video laryngoscopy or tracheostomy, may be necessary depending on cyst size and location [5]. Despite established guidelines, each case demands individualized planning due to anatomical variations and patient cooperation [6].

This case series highlights the anesthetic challenges and successful airway management strategies in three adult patients with vallecular cysts, emphasizing preoperative assessment, optimal sedation, and meticulous execution to prevent complications. By reviewing these cases, we aim to reinforce the importance of tailored airway approaches in similar high-risk scenarios.

## Case presentation

All patients were premedicated with glycopyrrolate (0.2 mg IM) to reduce secretions and midazolam (1 mg) with fentanyl (1 mcg/kg). Airway was anaesthetised topically with nebulization using 4 ml of 4% lignocaine and nasal packing with 2 ml of 2% adrenalized lignocaine gauze. Airway block includes bilateral superior laryngeal nerve block using 2 ml of 2% lignocaine and transtracheal block was done with 3 ml of 2% lignocaine. Pre-operatively low-dose midazolam (1-2 mg)+fentanyl (2 mcg/kg) for AFOI was given. Nasal decongestion (oxymetazoline drops) was used wherever necessary. Following AFOI, intraoperatively, patients were induced with propofol (2 mg/kg) and muscle relaxant vecuronium (0.1 mg/kg). The plane of anaesthesia maintained in all three cases with 50:50 mixture of oxygen and nitrous oxide along with sevoflurane (MAC 1.0). Intraoperative medications included dexamethasone 8 mg and paracetamol 1 g intravenously for anti-inflammatory and analgesic purposes.

Case 1

A 55-year-old male patient presented with complaints of progressive voice change and a throat-blocking sensation experienced while swallowing both solids and liquids, persisting for the past three months. He also reported a dry cough but denied fever, cold, or any systemic illnesses. On general examination, he was conscious, oriented, and afebrile, weighing 65 kg, with no clinical signs of pallor, clubbing, cyanosis, or lymphadenopathy. Systemic examination was unremarkable.

A high-resolution CT scan of the neck revealed a mildly enhancing polypoidal lesion measuring 16×24×32 mm arising from the left posterior oropharyngeal wall, displacing the tongue anteriorly and the epiglottis inferiorly. Cardiologic evaluation showed an ejection fraction of 67% with an interatrial septal aneurysm, deemed low risk for anesthesia. Pulmonary function tests indicated severe obstruction, classifying the patient as high risk by the pulmonologist.

Following intubation, intraoperative analgesia included intravenous dexmedetomidine (40 mcg) and fentanyl (50 mcg). The procedure, which involved marsupialization of the cyst, lasted approximately 90 minutes, with an estimated blood loss of 20 ml and 1200 ml of intravenous fluids administered. Postoperatively, the patient was reversed after confirming adequate respiratory effort and extubated without complications. He was observed for two days post-surgery and had an uneventful recovery.

Case 2

A 48-year-old male patient presented with a 15-day history of dysphagia and throat pain, along with dysphonia that had persisted for two months. He had no significant past medical history. On examination, he was alert, afebrile, and hemodynamically stable with a heart rate of 88 bpm, blood pressure of 110/80 mmHg, and oxygen saturation of 98% on room air. The airway assessment revealed a Mallampati class II with a three-finger mouth opening, unrestricted neck movement, and no dental anomalies.

**Figure 1:**
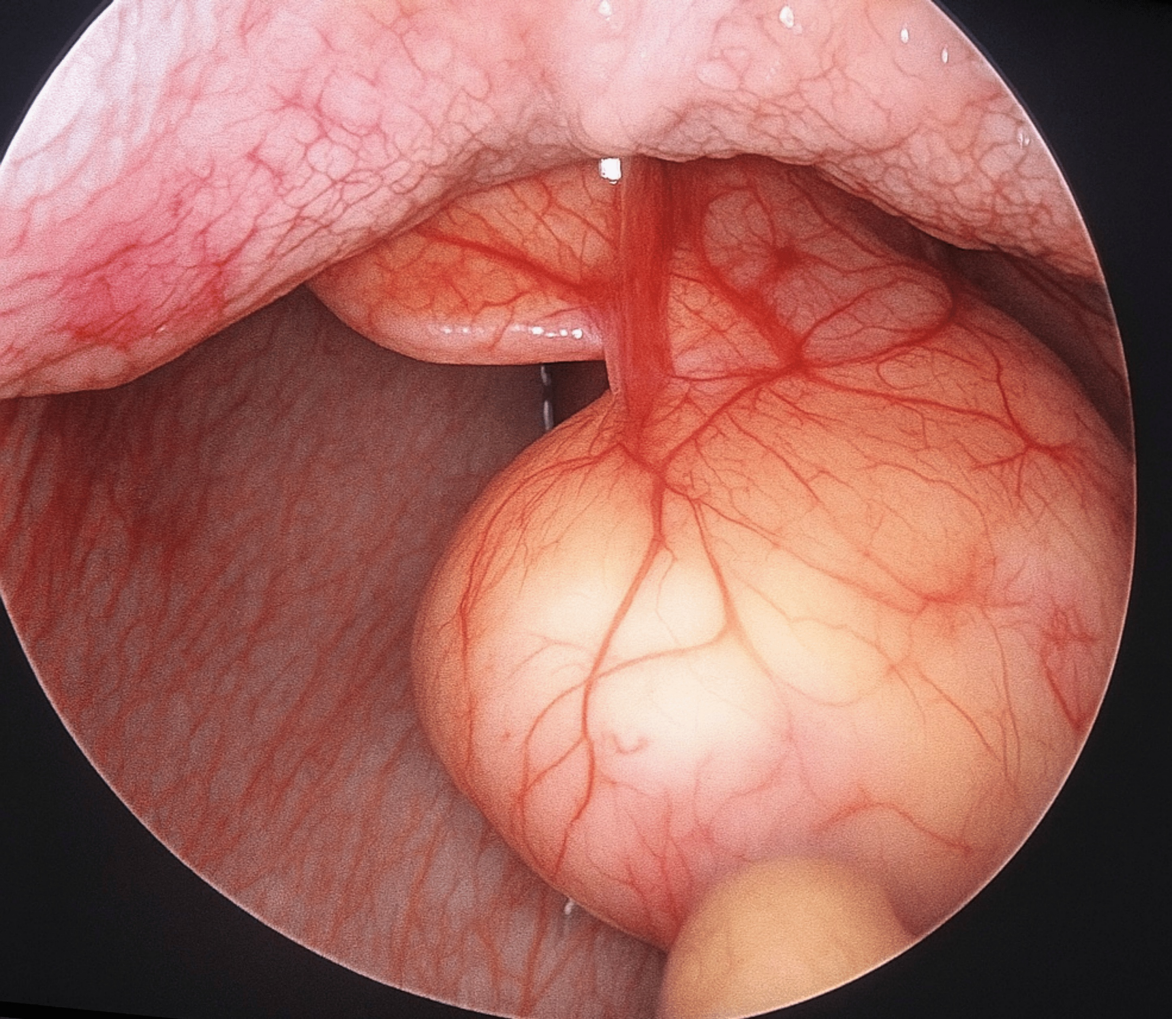
Vallecular cyst Endoscopic view through oral cavity showing intact cyst attached to epiglottis. Photograph by Dr. M. Dharani.

Video laryngoscopy showed a cystic lesion measuring approximately 4×3 cm arising from the left vallecula, attached posteriorly to the epiglottis, which appeared tubular .Contrast-enhanced CT of the neck confirmed a well-defined peripherally enhancing cystic lesion at the left vallecula as show in Figure 1.

Following AFOI, the patient underwent partial epiglottectomy uneventfully. The total duration of the procedure was 130 minutes. Postoperative recovery was smooth, and the patient was extubated after regaining adequate respiratory effort without complications.

Case 3

A 52-year-old male patient presented with a six-week history of a persistent globus sensation and mild difficulty swallowing solid food. He also reported intermittent hoarseness of voice but denied respiratory distress, stridor, fever, or systemic symptoms. On general examination, the patient was conscious, oriented, and afebrile, with a body weight of 72 kg. His vital signs were stable: heart rate 82 bpm, blood pressure 120/78 mmHg, respiratory rate 16/min, and SpO₂ 98% on room air. Airway examination revealed a Mallampati class III with a mouth opening of approximately 2.5 finger breadths. Neck mobility was full, and there were no loose teeth or other anticipated anatomical challenges. Cardiovascular, respiratory, and abdominal system evaluations were within normal limits.

A flexible nasopharyngoscopy revealed a smooth, cystic swelling originating from the right vallecular region, measuring approximately 3.5 cm in diameter, partially obscuring the view of the epiglottis. A contrast-enhanced CT scan of the neck confirmed a well-defined, fluid-density lesion consistent with a vallecular cyst.

Awake nasal fiberoptic intubation was successfully performed via the left nostril using a size 6.5 flexometallic endotracheal tube, which was secured after confirming bilateral breath sounds. The patient underwent endoscopic excision of the vallecular cyst without intraoperative complications. The procedure lasted approximately 75 minutes, with an estimated blood loss of 30 ml. The patient remained hemodynamically stable throughout surgery. He was extubated once spontaneous respiration and airway reflexes were adequate, following standard reversal with neostigmine and glycopyrrolate. Postoperative monitoring was uneventful, and the patient was discharged on postoperative day three with complete symptom resolution and no recurrence at follow-up.

**Table 1:** Case comparsion table ASA: American Society of Anesthesiologists; FMT: flexometallic tube.

Case	Demographic (Age, Gender, ASA)	Chief Complaints and Duration	Location of the Cyst	Airway Management	Outcome
Case 1	55-year-old male, ASA Grade 2	Voice change and throat-blocking sensation - 3 months; dry cough	Left posterior oropharyngeal wall	Awake nasal fiberoptic intubation with topical anaesthesia and blocks	Uneventful extubation; 2-day observation; complete recovery
Case 2	48-year-old male, ASA Grade 2	Dysphagia and throat pain - 15 days; dysphonia - 2 months	Left vallecula (attached to epiglottis)	Awake nasal fiberoptic intubation with topical anaesthesia and blocks	Smooth extubation; no complications
Case 3	52-year-old male, ASA Grade 2	Globus sensation - 6 weeks; difficulty swallowing solids; intermittent hoarseness	Right vallecula	Awake nasal fiberoptic intubation via left nostril (6.5 mm FMT) with topical anaesthesia and blocks	Uneventful extubation; discharged on day 3; complete symptom resolution

## Discussion

Vallecular cysts are rare, benign lesions arising from the mucous glands at the base of the tongue or epiglottis. While often asymptomatic, they can present significant challenges in airway management, particularly during anesthesia induction. This discussion delves into the complexities of managing vallecular cysts, emphasizing the importance of individualized approaches to ensure patient safety.

In adults, vallecular cysts may remain asymptomatic or present with nonspecific symptoms such as dysphagia, globus sensation, voice changes, or airway obstruction. The size and location of the cyst significantly influence the severity of symptoms. Flexible nasopharyngolaryngoscopy is a valuable diagnostic tool, allowing direct visualization of the cyst. Imaging studies, such as contrast-enhanced computed tomography (CT) or magnetic resonance imaging (MRI), provide detailed information about the cyst's size, extent, and relationship with surrounding structures.

The primary concern in patients with vallecular cysts is securing the airway without causing cyst rupture, which can lead to aspiration, laryngospasm, or complete airway obstruction. AFOI in sitting can be considered in very difficult cases. The risk is heightened during anesthesia induction when muscle relaxation and loss of protective reflexes occur. AFOI is considered the gold standard for managing anticipated difficult airways, as it allows for continuous patient cooperation and spontaneous ventilation [7]. However, AFOI requires patient cooperation and may not be feasible in all cases. The patient consent was obtained in view of the study.

Alternative techniques, such as video laryngoscopy, have been employed successfully. The King Vision® videolaryngoscope, for instance, has been used in cases where unexpected vallecular cysts were encountered, providing a clear view of the glottis and facilitating intubation [8]. In some situations, a tracheostomy may be necessary, especially when the cyst is large or obstructs the airway significantly.

A thorough preoperative assessment is crucial. Imaging studies help delineate the cyst's size and its relationship with surrounding structures. Preoperative airway evaluation, including Mallampati classification and neck mobility assessment, aids in anticipating potential difficulties. Premedication with anticholinergic agents like glycopyrrolate reduces secretions, facilitating airway visualization. Sedation should be carefully titrated to maintain spontaneous ventilation [7]. Topical anesthesia of the airway using lidocaine sprays or nebulization enhances patient comfort during awake intubation.

During surgery, maintaining hemodynamic stability and ensuring adequate oxygenation are paramount. The use of agents like sevoflurane for maintenance anesthesia, along with intravenous analgesics, provides a balanced anesthetic approach. Intraoperative monitoring should be vigilant to detect any signs of airway compromise promptly. Surgical excision of the cyst can be performed using various techniques, including cold steel instruments, electrocautery, or laser. The choice depends on the cyst's size, location, and the surgeon's expertise. Postoperative monitoring is essential to detect any complications early. Extubation should be approached cautiously, ensuring the patient has regained adequate spontaneous ventilation and airway reflexes. In the cases presented, patients were extubated uneventfully and monitored closely, leading to favorable outcomes.

Vallecular cysts are rare clinical entities in adults, with limited documented cases in the literature, making them an important subject for discussion in airway management [9]. Their rarity contributes to a lack of widespread clinical experience, increasing the potential for unexpected complications during anesthesia and surgery. The true incidence remains unclear, but case reports and small case series suggest they are an uncommon but high-stakes diagnosis, particularly when undetected prior to airway intervention.

The most critical concern with vallecular cysts is their propensity for rupture during airway manipulation, which can lead to catastrophic aspiration of cyst contents, resulting in laryngospasm, bronchospasm, or complete airway obstruction. The cyst wall is often thin and fragile, making it susceptible to trauma during laryngoscopy, intubation, or even during routine suctioning. Rupture can cause sudden flooding of the airway with mucoid or purulent material, leading to acute hypoxemia, pneumonia, or even respiratory arrest [4]. Given that many patients present with subtle symptoms, the cyst may remain undiagnosed until airway instrumentation precipitates an emergency, emphasizing the need for high clinical suspicion and preoperative imaging in patients with unexplained dysphagia or voice changes [5].

Additionally, the location of vallecular cysts near the epiglottis and laryngeal inlet increases the risk of mechanical obstruction during the induction of anesthesia. Loss of muscle tone from sedatives or neuromuscular blockers can cause the cyst to occlude the airway, leading to "can't intubate, can't oxygenate" (CICO) scenarios [6]. This risk is compounded in cases where the cyst is large (>3 cm), as it may distort anatomy and obscure the glottic view [7]. The combination of potential rupture and obstruction makes vallecular cysts one of the most hazardous benign lesions in airway management.

The high morbidity associated with mismanagement further underscores the importance of these cases. Reports describe instances where unrecognized cysts led to emergency tracheostomies, prolonged intensive care unit (ICU) stays, or even fatal outcomes due to aspiration or failed ventilation [3]. The Fourth National Audit Project (NAP4) highlighted that pharyngeal pathology, including cysts, significantly increases the risk of major airway complications, reinforcing the need for specialized techniques like AFOI and contingency planning [9].

Given these risks, multidisciplinary coordination between anesthesiologists, otolaryngologists, and radiologists is essential. Preoperative imaging (CT/MRI) and endoscopic evaluation should be routine in suspected cases to assess cyst size, location, and potential airway compromise [8]. Furthermore, simulation training and case-based learning can improve preparedness among anesthesia providers, as real-world exposure to such cases remains limited [10].

## Conclusions

Vallecular cysts, though uncommon, present significant anesthetic challenges due to their potential to obstruct the airway, hence securing airway with safe manner is primary anaesthetic goal. Adequate pre-op preparation of airway along with successful airway blocks facilitated AFOI in all our cases successfully.
